# Antimicrobial and Antibiofilm Activities of Sulfated Polysaccharides from Marine Algae against Dental Plaque Bacteria

**DOI:** 10.3390/md16090301

**Published:** 2018-08-27

**Authors:** Joon-Young Jun, Min-Jeong Jung, In-Hak Jeong, Koji Yamazaki, Yuji Kawai, Byoung-Mok Kim

**Affiliations:** 1 Division of Strategic Food Research, Korea Food Research Institute, Gangneung 25440, Korea; jjy791223@nate.com (J.-Y.J.); ooojmj@nate.com (M.-J.J.); 2 Department of Marine Food Science and Technology, Gangneung-Wonju National University, Gangneung 25457, Korea; ihjeong@gwnu.ac.kr; 3 Faculty of Fisheries Sciences, Hokkaido University, Hakodate 041-8611, Japan; yamasaki@fish.hokudai.ac.jp (K.Y.); kawai@fish.hokudai.ac.jp (Y.K.)

**Keywords:** dental plaque biofilm, dental caries, marine alga, sulfated polysaccharide, antimicrobial activity, monosaccharide, molecular weight

## Abstract

Dental plaque biofilms cause various dental diseases; therefore, inhibiting the growths of the dental plaque bacteria which produce biofilms can be a strategy for preventing dental disease. Certain sulfated polysaccharides from marine algae exert antimicrobial activities against human bacterial pathogens in addition to their physiological benefits. On the basis of these observations, the antimicrobial and antibiofilm activities of sulfated polysaccharides from different marine algae were evaluated against dental plaque bacteria. Among the sulfated polysaccharides, a fucoidan from *Fucus vesiculosus* showed notable antimicrobial activities against the selected dental plaque bacteria, including some foodborne pathogenic bacteria. The minimum inhibitory concentrations were of 125 to 1000 µg mL^−1^. Regarding the antibiofilm activity, the fucoidan at the concentrations of above 250 µg mL^−1^ completely suppressed the biofilm formations and planktonic cell growths of *Streptococcus mutans* and *S. sobrinus*. However, no eliminative effect on the completed biofilm was observed. The fucoidan consisted of almost fucose base polysaccharide containing approximately 14.0% sulfate content. The average molecular weight of the fucoidan was changed by heat treatment (121 °C for 15 min) and it affected the antimicrobial activity.

## 1. Introduction

Recently, marine algae have attracted increasing attention as a new source for bioactive compounds in addition to their nutritional importance, since the potential medicinal effects of algal constituents against various diseases or physiological concerns, such as anti-allergy, anticancer, antihypotension, anti-inflammation, anti-obesity, antioxidation, and antithrombosis effects, have been revealed [1,2]. In the terms of antimicrobial agents, marine algae have been globally tested, and various potential candidates, such as algal lectins, bromo-diterpenes, halogenated furanones, phlorotannins, and sesquiterpenes, have been found [3,4]. However, the industrial utilization of these candidates as antimicrobial agents or food preservatives is not common. This might be due to the infinitesimal recovery of such agents from the raw materials or the structural complexity for synthesis.

Marine algae contain large amounts of indigestible polysaccharides, such as agar, alginate, and carrageenan, which have been employed in dietary fibers as functional ingredients for human gut health or industrially utilized as colloidal materials to improve the rheological characteristics of food matrices [5]. Beside these utilizations, several studies demonstrated the potential of sulfated polysaccharides, such as fucoidan, ulvan, and carrageenan, as antimicrobial agents against a wide range of human bacterial pathogens [6,7,8,9,10]. In addition, these sulfated polysaccharides have been known to possess multiple bioactive properties [11]. However, the chemical properties of such sulfated polysaccharides vary with algal species, and as the bioactivity might be different according to their structure, molecular size, and sulfate amount [11,12], not only the screening of their antimicrobial properties, but also their chemical characterization are required.

In dental disease, dental plaque biofilms are known to cause dental caries, periodontitis and gingivitis, though the biofilms are also present on healthy teeth [10]. Dental caries is often associated with increases of acidogenic mutans streptococci and lactobacilli, which can metabolize sugars to acid and consequentially lead to the demineralization of enamel [13]. Biofilm can be defined as a community of certain microorganisms attached to a surface, which are generally encapsulated and protected by an extracellular matrix composed of various biopolymers. Therefore, the elimination of microbial biofilms is not easy, and its presence displays antimicrobial resistance [14,15]. Inhibiting the initial biofilm formation of dental plaque bacteria can be a strategy for preventing dental caries.

In this connection, the antimicrobial activities of different sulfated polysaccharides from marine algae were screened against dental plaque bacteria, including foodborne pathogenic bacteria. For a potential sulfated polysaccharide, its antibiofilm activity against selected dental plaque bacteria was evaluated, and the chemical property was also investigated.

## 2. Results and Discussion

### 2.1. Isolation Yield of Sulfated Polysaccharides

In total, eight polysaccharides from different marine algae (five brown algae, two green algae, and one red alga, which are edible) were isolated (Table 1). The isolation yield was the highest in the red alga *Grateloupia flilicina* (28.5%, dry), and the others ranged from 2.8 to 5.7% (dry). Although there was no information for the isolation yields of the four commercial fucoidans (F85, F95, M85, and U95) used in this study, it has been reported that fucoidans were yielded in the rages of 3.9 to 9.5% from different brown algae [16]. In general, the cell walls of brown algae are composed of celluloses, alginates, and fucoidans in an average weight ratio of 1:3:1 (in the order mentioned) [17]. These polysaccharides are mostly indigestible and account for 40.0 to 50.7% (dry) of the whole algae [18]. On the basis of these ratios, the biomass of fucoidan in brown algae can be estimated to be ca. 8.0 to 10.1% (dry), but the average yield with the acid extraction method [19,20] employed in this study was comparatively low.

### 2.2. Chemical Properties of Sulfated Polysaccharides

To specify the fundamental chemical properties of sulfated polysaccharides, the total sugar, mineral, and sulfate contents were determined (Table 1). The chemical properties of sulfated polysaccharides vary with algal species, but they contain a primary monosaccharide and sulfates [21]. However, the polysaccharide from *Codium fragile* prepared with the water extraction method [22] did not contain sulfate. The total sugar contents of the four commercial fucoidans ranged from 83.5 to 90.5%, while those of the sulfated polysaccharides isolated in this study, except for that from *C. fragile*, ranged from 63.0 to 75.1%. All the isolated sulfated polysaccharides were dialyzed with deionized water (DW), but significant amounts of minerals (2.2–7.6%) remained. At a concentration of 2.5 mg mL^−1^, the pH values of fucoidans F85 and M85 were acidic (pH 5.31 and 4.93, respectively), while those of all the isolated sulfated polysaccharides and a commercial fucoidan U95 were in the ranges of 6.05 to 7.81. In contrast, fucoidan F95 exhibited a basic pH (9.46), though it contained 7.5 ± 2.1% sulfate. According to the algal classification based on pigments, the sulfate content was higher in the brown algae (6.4–14.0%, including commercial fucoidans) than in those from the green and red algae (0–3.0%).

### 2.3. Antimicrobial Screening of Sulfated Polysaccharides

The antimicrobial activities of the sulfated polysaccharides from 11 species of marine algae (including four commercial fucoidans) were screened against six foodborne pathogenic bacteria, eight dental plaque bacteria, and two lactic acid bacteria by determining the minimum inhibitory concentration (MIC) (Table 2). Among the sulfated polysaccharides, only the fucoidan F85 from *Fucus vesiculosus* showed growth inhibitory effects against all the dental plaque bacteria tested in this study, with MICs of 125 to 1000 µg mL^−1^; *S. mutans* KCTC 5458 was the most sensitive among the tested bacterial strains. In addition, the fucoidan F85 inhibited the growths of *Listeria monocytogenes* KCTC 13,064 (250 µg mL^−1^), *Staphylococcus aureus* KCTC 3881 (500 µg mL^−1^), and the two lactic acid bacteria (500 µg mL^−1^ in both), but not of the Gram-negative bacteria. In contrast, the fucoidan F95 did not inhibit growths of any bacterial strains, though it is also originated from the *F. vesiculosus*. Beside the fucoidan F85, the two sulfated polysaccharides from brown algae *Undaria pinnatifida* and *Kjellmaniella crassifolia* had growth inhibitory effects against *Salmonella typhimurium* at a concentration of 1000 µg mL^−1^ (in both). The MICs of ampicillin (as a standard antimicrobial agent) were in the ranges of 0.8 to 12.5 µg mL^−1^ for the dental plaque bacteria.

Although there was not much reports that addressed the antimicrobial activity of sulfated polysaccharides from marine algae, especially regarding dental bacterial pathogens, a similar result was found in a study conducted by Lee et al. [10], a fucoidan exerted potential antimicrobial activities against several cariogenic *Streptococcus* sp. (MICs, 250–500 µg mL^−1^; minimum bactericidal concentrations (MBCs), 500–1000 µg mL^−1^) and the periodontopathogenic bacteria *Actinobacillus actinomycetemcomitans*, *Fusobacterium nucleatum*, *Prevotella intermedia*, and *Porphylomonas gingivalis* (MICs, 125–500 µg mL^−1^; MBCs, 250–1000 µg mL^−1^). Regarding other human bacterial pathogens, Marudhupandi and Kumar [9] reported that a fucoidan from a brown alga, *Sargassum wightii*, possessed a broad antibacterial spectrum against *Escherichia coli*, *Klebsiella pneumoniae*, *Vibrio cholerae*, *Proteus proteus*, *Shigella sonnie*, *Pseudomonas aeruginosa*, *S. typhimurium*, and *Klebsiella* sp., with MICs of 31.25 to 250 µg mL^−1^. From the green alga *Chaetomorpha aerea*, a sulfated polysaccharide was isolated, which showed selective antibacterial activities against the Gram-positive bacteria *Bacillus subtilis*, *Micrococcus luteus*, and *S. aureus* [8]. Yamashita et al. [6] studied the effects of several dietary polysaccharides originated from marine algae and land plants on the growth of some foodborne bacteria. In their report, carrageenans (λ, γ and κ) at 2500 µg mL^−1^ had bacteriostatic effects against *S. enteritidis*, *S. typhimurium*, *Vibrio mimicus*, *Aeromonas hydrophila*, *E. coli* (enterotoxigenic), and *S. aureus*. However, no effect of the γ-carrageenan at even a high-level dose of 5000 µg mL^−1^ was observed in all the tested bacterial strains in this study (data not shown).

### 2.4. Antibiofilm Screening of Fucoidan F85 against Selected Dental Plaque Bacteria

**Figure 1:**
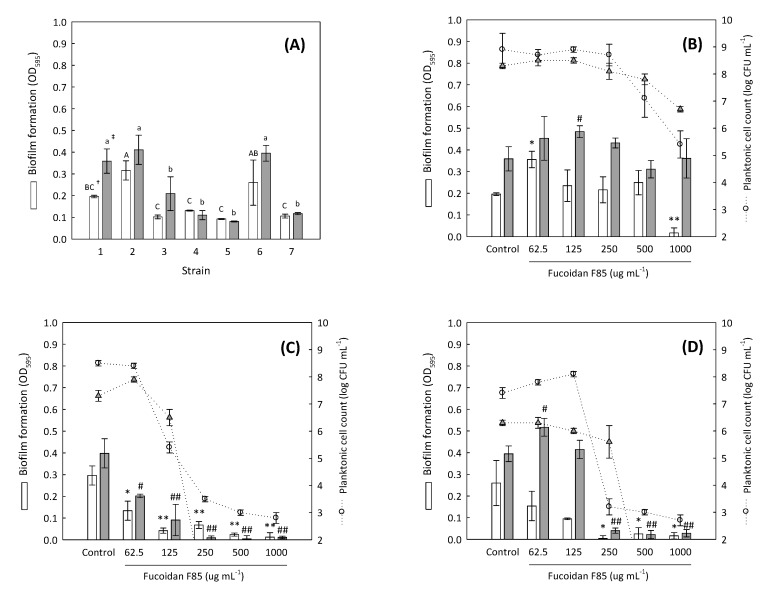
Biofilm formation of several dental plaque bacteria in brain heart infusion (BHI) broth for 48 h and the antibiofilm activities of fucoidan F85 against the selected dental plaque bacteria. (**A**) biofilm formation of several dental plaque bacteria; (**B**) *E. faecalis* KCTC 5289; (**C**) *S. mutans* KCTC 5458; (**D**) *S. sobrinus* KCTC 5809. □, biofilm formation (24 h); ■, biofilm formation (48 h); ○, planktonic cell count (24 h); ▲, planktonic cell count (48 h). 1, *E. faecalis* KCTC 5289; 2, *S. mutans* KCTC 5458; 3, *S. mutans* KCCM 40105; 4, *S. oralis* KCCM 41567; 5, *S. sanguinis* KCTC 5643; 6, *S. sobrinus* KCTC5809; 7, *S. sobrinus* KCCM 11898. Data expressed as the mean ± SD in triplicate. The initial cell concentration was ca. 5 log CFU mL^−1^. ^†^ The different capital letters indicate significantly different values in 24 h (*p* < 0.05, ANOVA). ^‡^ The different small letters indicate significantly different values in 48 h (*p* < 0.05, ANOVA). * *p* < 0.05 versus growth control in 24 h (independent *t*-test), ** *p* < 0.01 versus growth control in 24 h (independent *t*-test), ^#^ *p* < 0.05 versus growth control in 48 h (independent *t*-test), and ^##^ *p* < 0.01 versus growth control in 48 h (independent *t*-test).

In dental plaque, there are over 600 species of bacteria and archaea, but approximately 50% of them are currently unculturable because of the limited growing conditions and nutrients [13]. For this reason, the reproduction of an artificial dental plaque microbiota in vitro is not easy, and only a single or small number of combined strains can be tested. The fucoidan F85 was selected for further experiment, and the biofilm formative abilities of different *Streptococcus* sp. (six strains) including one *Enterococcus* sp. were measured to select suitable test strains for the determination of antibiofilm activity. All the strains were originated from human dental plaques and are easily culturable. At both incubation times of 24 h and 48 h, distinct biofilm formations were found in the strains *Enterococcus faecalis* KCTC 5289, *S. mutans* KCTC 5458, and *S. sobrinus* KCTC 5809, and their biofilm formations were significantly higher than those of the other strains (*p* < 0.05) (Figure 1A).

Thus, the three dental plaque bacteria were selected as the test strains and the antibiofilm activity of fucoidan F85 was evaluated. For *E. faecalis* KCTC 5289 associated biofilm (Figure 1B), fucoidan F85 at a concentration of 1000 µg mL^−1^ suppressed approximately 91.3% of biofilm formation for 24 h compared to growth control, and this was accompanied with the growth inhibition of planktonic cells. However, the suppressive effect was lost in 48 h. For *S. mutans* KCTC 5458 (Figure 1C), the antibiofilm activity was observed in all the tested concentrations (62.5–1000 µg mL^−1^), which tended to be concentration-dependent. In particular, the biofilm formation and planktonic cells were completely suppressed for 48 h above the concentration of 250 µg mL^−1^. For *S. sobrinus* KCTC 5809 (Figure 1D), fucoidan F85 had a similar activity as that exhibited for *S. mutans* in 24 h, whereas the activities of the fucoidan F85 at the concentrations of 62.5 and 125 µg mL^−1^ were lost in 48 h. In summary, the fucoidan F85 at the concentrations of above 250 µg mL^−1^ completely suppressed both the biofilm formation and planktonic cell growths of *S. mutans* and *S. sobrinus*. 

### 2.5. Time-Coursed Inhibitory Effect of Fucoidan F85 on the Growth of S. mutans

Most antimicrobial agents from marine algae have been identified as primary phenolic compounds and sulfated polysaccharides. Although the structures of the phenolic compounds present in marine algae and terrestrial plants are different, it has been suggested that the antimicrobial action might be associated with their permeable effects into the bacterial cell membrane; the phenolic compounds permit and cause widening of the pores of the bacterial cell membrane, and consequently, the loss of intracellular macromolecules such as nucleotides and proteins may occur [23]. In contrast, the antimicrobial action of sulfated polysaccharides has not been clearly revealed. There are two theories explaining the antimicrobial action of sulfated polysaccharides (carrageenans) proposed by Yamashita et al. [6]: one suggests the binding of the sulfated polysaccharides on the bacterial surface, although in an experiment using ^3^H-carrageenan, no binding for *S. enteritidis* was observed; the other suggests the trapping of nutrients or cationic minerals induced by the sulfated polysaccharides, which can reduce the bioavailability of nutrients.

**Figure 2:**
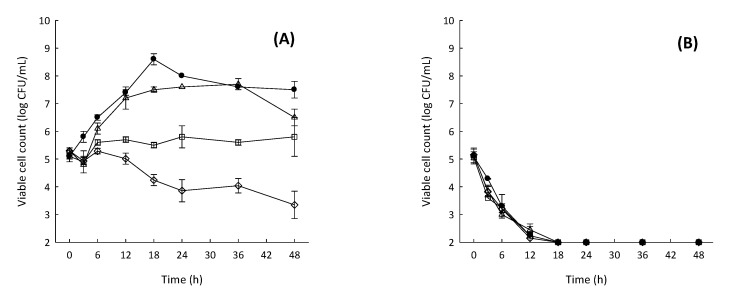
Time-coursed growth inhibitory effect of fucoidan F85 against *S. mutans* KCTC 5458 in BHI broth and phosphate buffered saline (PBS, pH 7.2) for 48 h. (A) BHI broth; (B) PBS. ●, growth control; Δ, 62.5 µg/mL of fucoidan F85 (MIC/2); □, 125 µg/mL of fucoidan F85 (MIC); ◊, 250 µg/mL of fucoidan F85 (2MIC). Data expressed as the mean ± SD in triplicate. The initial cell concentration was ca. 5 log CFU mL^−1^.

To understand the antimicrobial action of the fucoidan F85 based on the hypothesis proposed by Yamashita et al. [6], the time-coursed inhibitory effect against *S. mutans* was measured for 48 h under nutrient presence (BHI broth) or absence (phosphate buffered saline, PBS, pH 7.2) (Figure 2). The treated concentrations of fucoidan F85 were of 0 to 250 µg mL^−1^. In BHI broth, the growth control increased from ca. 5.2 log CFU mL^−1^ (the initial cell count) to ca. 8.6 log CFU mL^−1^ for 18 h and retained until 48 h with an approximately 1 log cycle of decrease. In contrast, there was no increase in the *S. mutans* treated with fucoidan at 125 µg mL^−1^, and the *S. mutans* treated with fucoidan F85 at 250 µg mL^−1^ showed a constant decrease to ca 3.3 log CFU mL^−1^ for 48 h. In PBS, all the experimental groups treated with fucoidan F85 and growth control showed a similar tendency; the *S. mutans* decreased consistently and reached a detection limit of ≤2 log CFU mL^−1^ within 18 h. As no rapid cell decreases in all the *S. mutans* treated with fucoidan F85 were observed in BHI broth and the cell decrease of the *S. mutans* treated with fucoidan F85 was similar with the growth control in PBS; it was suggested that the fucoidan F85 might not possess a direct killing effect

### 2.6. Inhibitory Effect of Fucoidan F85 according to the Treatment Time on the Biofilm Formation of S. mutans

As is well described in a review of Huang et al. [24], oral biofilm formation is classified into four stages (pellicle formation, initial adhesion, maturation, and dispersion), and *Actinomyces* sp., *Streptococcus* sp., *Haemophilus* sp., *Capnocytophaga* sp., *Veillonella* sp., and *Neisseria* sp. have been known to engage in the initial adhesion stage to tooth surface pellicle. In the process of bacterial biofilm formation, the initial adhesion rapidly occurred by a weak electrostatic and Van der Waals’ forces; in a short time, gene expression is modified, and formation of the biofilm begins with the physical adhesion of bacterial cells to the material surface by complex polysaccharides [25]. As the bacterial cells are encapsulated and protected by an extracellular matrix composed of various biopolymers, the elimination of microbial biofilms is not easy [14,15].

**Figure 3:**
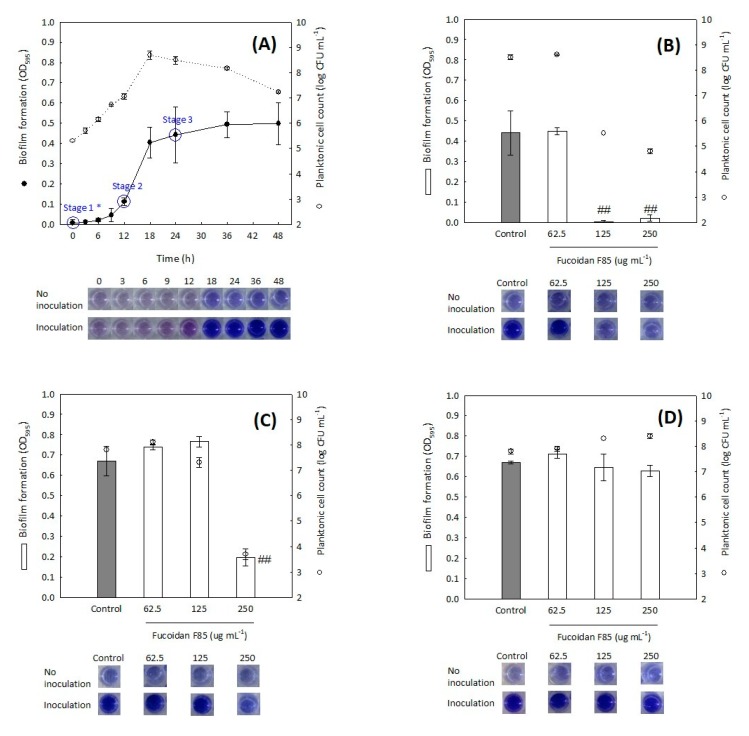
Inhibitory effect of fucoidan F85 according to the treatment time on the biofilm formation of *S. mutans* KCTC 5458. (**A**) time-coursed biofilm formation of *S. mutans* KCTC 5458 for 48 h; (**B**) antibiofilm activity of fucoidan F85 when treated at stage 1 (0 h); (**C**) antibiofilm activity of fucoidan F85 when treated at stage 2 (12 h); (**D**) antibiofilm activity of fucoidan F85 when treated at stage 3 (24 h). ●, biofilm formation; ○, planktonic cell count; □ and ■, biofilm formation (after 24 h). Data expressed as the mean ± SD in triplicate. The initial cell concentration was ca. 5 log CFU mL^−1^. * treatment time: stage 1, 0 h; stage 2, 12 h; stage 3, 24 h. ^##^ *p* < 0.01 versus growth control (independent *t*-test).

To investigate the change in the antibiofilm activity of fucoidan F85 according to the treatment time, fucoidan F85 was treated at different times, which were set by the determination of the time-coursed biofilm formation of *S. mutans* (Figure 3A). The biofilm formation was clearly distinguished from 12 h of incubation and reached nearly a maximal level at 24 h. The planktonic cells proliferated after the inoculation immediately, while the biofilm formation seemed to have a lag phase for 6 h. The process of the biofilm formation for 48 h in BHI broth might be divided into the three stages: the initial stage (stage 1, 0 h), developmental stage (stage 2, 12 h), and complete stage (stage 3, 24 h). The results for inhibitory effects of fucoidan F85 when treated at each the three stages are given in Figure 3B–D. In the case of the treatment at the stage 1, the fucoidan F85 at above 125 µg mL^−1^ suppressed both the biofilm formation and planktonic cell growth, while the suppressive effect greatly diminished when the fucoidan F85 was treated at the stage 2, and even no effect was observed in the *S. mutans* treated at the stage 3, considering that the completed biofilm could not be eliminated by the fucoidan treatment.

### 2.7. Composite Monosaccharides and Molecular Weight Estimation

Between the two fucoidans (F85 and F95) from *F. vesiculosus*, only fucoidan F85 possessed antimicrobial activity. If the molecular difference between the two fucoidans is revealed, it might give an insight for understanding the relation between the molecular characteristics and the antimicrobial activity. As fucoidans were mostly composed of carbohydrates, their composite monosaccharides were determined. In the composite monosaccharides, fucose was dominant (above 87.4%) in both fucoidans, and galactose, mannose, and xylose were detected in both as well (Table 3). Apart from the fact that fucoidan F85 contained a lower amount of glucose, there was not much difference in the contents of the composite monosaccharides between the two compounds.

Li et al. [26] described the structure of the fucoidan from *F. vesiculosus*, which is composed of α-(1,3)-linked fucose with sulfate groups substituted at the C-4 position on some of the fucose residues. The electrical properties of the fucoidan could be associated with the sulfate groups and it might affect the antimicrobial activity. However, in our study, the fucoidan F95 did not possess any antimicrobial activity, though it contained ca 7.5% sulfate content. Furthermore, in a preliminary experiment, the antimicrobial activity of fucoidan F85 decreased without heat treatment (121 °C for 15 min); the MIC for *S. mutans* was determined to be 1000 µg mL^−1^ (data not shown).

For this reason, the change in the molecular characteristic of the fucoidan F85 by heat treatment was expected and the molecular weights of the two fucoidans, with or without heat treatment, were estimated by gel permeation chromatography (Table 4 and Supplementary Information). The weight-average molar masses (Mws) of the fucoidan F85 and F95 without heat treatment were estimated to be ca. 74,214 g mol^−1^ and 62,080 g mol^−1^, respectively. After the heat treatment, the Mw of fucoidan F95 was almost unchanged, whereas that of fucoidan F85 notably decreased to ca. 13,875 g mol^−1^. As we found, not only sulfate groups, but also the Mws of sulfate polysaccharides, might play crucial roles in their antimicrobial activities.

## 3. Materials and Methods

### 3.1. Materials

Five brown algae (*Hizikia fusiforme*, *Kjellmaniella crassifolia*, *Laminaria japonica*, *Sargassum honeri*, and *Undaria pinnatifida*), two green algae (*Capsosiphon fulvescens* and *Codium fragile*) and one red alga (*Grateloupia flilicina*) that are edible and very popular, were purchased in a dried state from the Jumunjin fishery market (Gangneung, Korea), were used for isolation of sulfated polysaccharide in this study. The algae were ground, sieved on 40 mesh (≤350 µm), and stored at −20 °C before use. Four commercial fucoidans, crude fucoidan from *Fucus vesiculosus* (F85, purity: 85%), purified fucoidan from *Fucus vesiculosus* (F95, purity: 95%), crude fucoidan from *Macrocytis pyrifera* (M85, purity: 85%) and purified fucoidan from *U. pinnatifida* (U95, purity: 95%), were obtained from Sigma-Aldrich Inc. (St. Louis, MO, USA). A γ-carrageenan was purchased from FUJIFILM Wako Pure Chemical Co. (Osaka, Japan).

### 3.2. Isolation of Sulfated Polysaccharide

Two methods were employed for the isolation of crude sulfated polysaccharides according to algae classification based on pigments. For brown algae, the isolation was conducted according to the methods recommended by Koo et al. and Kim et al. [19,20]. Briefly, a 100 g sample of each algal powder was treated with 85% methanol for 2 h using a reflux system (at boiling point) and centrifuged at 7500× *g* for 15 min to remove lipid components and low-molecular-weight compounds. The treatment was replicated three times and the precipitates were collected. The precipitate was extracted with 1000 mL of diluted HCl (pH 2) at 65 °C for 1 h and centrifuged at 7500× *g* for 15 min. The pH of the supernatant was adjusted to 7 with NaOH. The acid extraction for the residue was replicated three times in total. The collected supernatant was concentrated, and 10% CaCl_2_ was added until a precipitate was formed. After centrifugation (7500× *g*, 15 min), the supernatant was dialyzed (Mw, 4500 cut-off) with DW for 12 h, transferred to 75% ethanol, and left standing for 12 h. After centrifugation at 10,000× *g* for 10 min, the precipitate was lyophilized and weighed. For green and red algae, the isolation was conducted according to the methods recommended by Cho et al. [22].

### 3.3. Determination of pH, Total Sugar, and Sulfate Contents

For the pH determination, each 12.5 mg of the sulfated polysaccharides was dissolved in 5 mL DW, and then the pH was determined using a pH meter. The concentration was based on their solubilities (visible turbidity). The total sugar content was measured according to an anthrone-sulfuric acid method, using glucose as a standard [27]. The sulfate content was determined according to a BaCl_2_ gelatin method after acid hydrolyzation of the sample using K_2_SO_4_ as a standard [28].

### 3.4. Microorganisms and Culturing Condition

The bacterial strains used in this study were as follows: foodborne pathogenic bacteria *Bacillus cereus* KCTC 3624, *Listeria monocytogenes* KCTC 13064, *Staphylococcus aureus* subsp. *aureus* KCTC 3881, *Aeromonas hydrophila* subsp. *hydrophila* KCTC 2358, *Escherichia coli* KCTC 2441, and *Salmonella typhimurium* KCCM 11862. They were maintained in cryogenic bead vials (Microbank^TM^; Pro-Lab Diagnostics Inc., Toronto, ON, Canada) at −70 °C and activated in brain heart infusion (BHI) broth (Difco; Becton Dickinson, Spark, MD, USA) at 35 °C in an aerobic atmosphere condition for 16–20 h before use; dental plaque bacteria *Enterococcus faecalis* KCTC 5289, *Streptococcus mutans* KCTC 5458, *Streptococcus mutans KCCM 40105*, *Streptococcus oralis* KCCM 41567, *Streptococcus sobrinus* KCTC 5809, *Streptococcus sobrinus KCCM 11,898*, and *Streptococcus sanguinis* KCTC 5643 were maintained in cryogenic bead vials at −70 °C and activated in BHI broth at 35 °C in a 20% CO_2_ atmosphere condition for 16–20 h; lactic acid bacteria *Lactobacillus acidophilus* KCTC 3164 and *Streptococcus thermophilus* KCTC 3658 were maintained in cryogenic bead vials at −70 °C and activated in de Man Rogosa and Sharpe (MRS) agar (Difco) at 35 °C in a 20% CO_2_ atmosphere condition for 16–20 h.

### 3.5. Minimum Inhibitory Concentration

The MICs of the sulfated polysaccharides for the foodborne pathogenic and dental plaque bacteria were determined according to the broth micro-dilution method guided by the Clinical and Laboratory Standards Institute (CLSI) [29], with slight modification. All samples were tested at the final concentrations of 15.6–1000.0 µg mL^−1^ (double dilution) in BHI broth after sterilization (121 °C, 15 min), and the tested bacterial cell concentration was ca. 5 × 10^5^ CFU mL^−1^. The incubation was conducted at 35 °C under a 20% CO_2_ atmosphere condition for 24 h. The MIC was read as the lowest concentration of the tested sample that inhibited visible bacterial growth. Ampicillin (Sigma-Aldrich Inc.) at the final concentrations of 0.8–50.0 µg mL^−1^ (double dilution) were used as standard antibiotic.

### 3.6. Antibiofilm Activity and Planktonic Cell Count

The biofilm formations of seven dental plaque bacteria (*E. faecalis* KCTC 5289, *S. mutans* KCTC 5458, *S. mutans* KCCM 40105, *S. oralis* KCCM 41567, *S. sanguinis* KCTC 5643, *S. sobrinus* KCTC 5809, and *S. sobrinus* KCCM 11898) and the antibiofilm activities of fucoidan F85 against selected bacteria (*E. faecalis* KCTC 5289, *S. mutans* KCTC 5458, and *S. sobrinus* KCTC 5809) were measured according to the method recommended by O’Toole [30], with slight modifications. Briefly, a 50 µL overnight culture of the bacteria (a final cell concentration of ca. 5 log CFU mL^−1^) was inoculated into the 150 µL BHI broth prepared in a flat bottom 96-well microplate (noncoated polystyrene) and incubated at 35 °C under a 20% CO_2_ atmosphere condition for either 24 h or 48 h without shaking; only when testing the antibiofilm activity, fucoidan F85 was added in the BHI broth at the final concentrations of 0, 62.5, 125, 250, and 1000 µg mL^−1^. After incubation, 100 µL of the broth culture containing planktonic cells was carefully taken and counted using BHI agar plates (35 °C in 20% CO_2_ atmosphere condition, 24 h). The rest of the broth was discarded. The biofilm, the community of the attached cells to the well surface, was stained with 200 µL of 0.1% crystal violet solution for 15 min, and then it was discarded and rinsed with sterile DW twice. The stained biofilm was recovered with 200 µL of 30% acetic acid and measured at 595 nm.

### 3.7. Time-Coursed Inhibitory Effect of Fucoidan F85 against S. mutans

The inhibitory effect of fucoidan F85 on the growth of *S. mutans* KCTC 5458 for 48 h were measured with the plate count method [4]. The fucoidan F85 was separately diluted with BHI broth or PBS (pH 7.2) to the final concentrations of 0–250 µg mL^−1^ each. The overnight culture (BHI, at 35 °C for 20 h) was inoculated to a cell concentration of 10^5^ CFU mL^−1^ and incubated at 35 °C under a 20% CO_2_ atmosphere condition for 48 h. During the incubation, each of the cultures at 0, 3, 6, 12, 18, 24, 36, and 48 h was collected and counted using BHI agar plate after the incubation at 35 °C under a 20% CO_2_ atmosphere condition for 24 h. The 25 to 250 colonies were counted and expressed as the logarithmic number of colony forming units per gram sample.

### 3.8. Inhibitory Effect of Fucoidan F85 According to the Treatment Time on the Biofilm Formation of S. Mutans

To investigate the change in the antibiofilm activity of fucoidan F85 according to the treatment time, fucoidan F85 was treated at different times, which were set by the determination of the time-coursed biofilm formation of *S. mutans* KCTC 5458 for 48 h. The biofilm formation, antibiofilm activity, and planktonic cell count were measured by the methods as mentioned above. The fucoidan F85 (at the final concentrations of 0, 62.5, 125, and 250 µg mL^−1^ each) was separately treated either at 0, 12, or 24 h and incubated for 24 h. For the treatments at 12 h and 24 h, the broth culture was discarded and the fresh BHI broth containing fucoidan F85 at each the concentrations was added and mixed.

### 3.9. Composite Monosaccharides

The composite monosaccharides in fucoidans F85 and F95 were quantitatively measured using high-performance anion exchange chromatography equipped with a pulsed amperometric detector (Dionex ICS-5000; Thermo Fisher Scientific, Waltham, MA, USA). Before analysis, a 10 mg sample was separately hydrolyzed with 100 µL of 72% H_2_SO_4_ at 30 °C for 2 h, further treated at 121 °C for 1 h with addition of 2.8 mL DW, and filtrated using a 0.2 µm polytetrafluoroethylene syringe filter unit. The analysis was conducted with a Dionex CarboPac PA-1 column (10 µm, 4 × 250 mm, Thermo Fisher Scientific). A 20 µL of the sample was injected and eluted with 18 mM NaOH at a flow rate of 1 mL min^−1^. The monosaccharides were identified and calculated by comparison with the retention times and areas of eight monosaccharide standards (fucose, rhamnose, arabinose, galactose, glucose, mannose, xylose, and fructose) with three points of the external standard method.

### 3.10. Molecular Weight

The Mws of fucoidans F85 and F95 were analyzed using gel permeation chromatography (Agilent 1260 infinity series; Agilent technologies, Santa Clara, CA, USA) equipped with a refractive index detector. To elucidate the change in the Mw by heat treatment, fucoidans F85 and F95 were diluted with DW at a concentration of 2.5 mg mL^−1^ and heat-treated at 121 °C for 15 min. Then, 100 µL of the treated sample was injected after filtration using a 0.45 µm nylon filter unit and eluted with 0.2 M sodium phosphate buffer (pH 7.0) at a flow rate of 1 mL min^−1^. Two Tskgel GMPWxl columns (13 µm, 7.8 × 300 mm, Sigma-Aldrich Inc.) were used and maintained at 40 °C. Data acquisition was performed using Cirrus GPC software and pullulan (180 to 642,000 Mw) was used as a standard to calibrate the column (Supplementary Information).

### 3.11. Statistical Analysis

All data, except for the pHs, MICs, and Mws of the sulfated polysaccharides, were expressed as the mean ± standard deviation (SD) in triplicate. The values for the biofilm formation of several dental plaque bacteria were statistically assessed with a one-way ANOVA test; a significant difference (*p* < 0.05) between the means was identified by the least significant difference and Tukey’s test using SPSS (IBM, Armonk, NY, USA). The values for the antibiofilm activities of fucoidan F85 against selected dental plaque bacteria were assessed by independent two-sample *t*-tests; the significance level between the fucoidan F85 dose-levels and the control was set at *p* < 0.05.

## 4. Conclusions

In the present study, the antimicrobial and antibiofilm activities of sulfated polysaccharides from different marine algae were evaluated against dental plaque bacteria. Among the sulfated polysaccharides, the fucoidan F85 possessed notable antimicrobial activities against the selected dental plaque bacteria with MICs of 125 to 1000 µg mL^−1^. In particular, the fucoidan F85 at the concentrations of above 250 µg mL^−1^ completely suppressed the biofilm formations and planktonic cell growths of *S mutans* and *S. sobrinus*, suggesting that the fucoidan F85 might be a potential in development of antibiofilm agent capable of inhibiting the biofilm formations of the two dental plaque bacteria. The chemical investigation revealed that the fucoidan F85 consisted of almost fucose base polysaccharide containing approximately 14.0% sulfate content. The average molecular weight of the fucoidan F85 was changed by the heat treatment (121 °C for 15 min) and it affected the antimicrobial activity.

## Figures and Tables

**Table 1:** Yield and chemical properties of sulfated polysaccharides according to algal species.

Origin	Yield		Total Sugar		Mineral		pH ^†^	Sulfate	
	(%, dry)		(%, dry)		(%, dry)			(%, dry)	
Brown algae									
*Fucus vesiculosus* (F85) *	Nt	^‡^	83.5	±1.9	2.2	±0.6	5.31	14.0	±2.7
*Fucus vesiculosus* (F95) *	Nt		90.5	±1.2	Nt		9.46	7.5	±2.1
*Macrocytis pyrifera* (M85) *	Nt		82.1	±2.3	Nt		4.93	11.4	±0.4
*Undaria pinnatifida* (U95) *	Nt		89.5	±1.2	Nt		6.05	10.5	±2.1
*Hizikia fusiforme*	3.7	±0.6	65.2	±0.7	6.8	±3.1	7.14	6.5	±1.4
*Kjellmaniella crassifolia*	3.0	±0.4	75.1	±2.1	6.9	±0.2	7.81	8.6	±2.3
*Laminaria japonica*	2.8	±0.3	62.3	±3.2	7.1	±0.4	7.73	6.4	±0.6
*Sargassum honeri*	3.5	±1.1	63.5	±0.4	6.3	±0.3	7.43	5.2	±1.7
*Undaria pinnatifida*	2.9	±0.3	63.0	±0.3	7.6	±0.6	7.71	7.8	±1.4
Green algae									
*Capsosiphon fulvescens*	4.5	±1.2	67.1	±1.3	5.2	±0.3	7.65	2.5	±0.6
*Codium fragile*	5.7	±0.4	58.2	±1.1	11.4	±0.8	7.11	Nd	^§^
Red alga									
*Grateloupia flilicina*	28.5	±0.9	65.1	±3.7	3.8	±0.4	6.52	3.0	±1.2

Data, except for pH, expressed as the mean ± SD, in triplicate. * Commercial fucoidans; the numbers in round brackets indicate the purity of the fucoidan. ^†^ The samples were measured at a concentration of 2.5 mg mL^−1^ in DW. ^‡^ Not tested. ^§^ Not detected.

**Table 2:** Minimum inhibitory concentrations (µg mL^−1^) of algal sulfated polysaccharides against pathogenic bacteria and lactic acid bacteria.

Bacterial Strain	1	2	3	4	5	6	7	8	9	10	11	12	13	14
Foodborne pathogenic bacteria														
*Bacillus cereus* KCTC 3624	NI *	NI	NI	NI	NI	NI	NI	NI	NI	NI	NI	NI	NI	25.0
*Listeria monocytogenes* KCTC 13064	250	NI	NI	NI	NI	NI	NI	NI	NI	NI	NI	NI	NI	3.1
*Staphylococcus aureus* subsp. *aureus* KCTC 3881	500	NI	NI	NI	NI	NI	NI	NI	NI	NI	NI	NI	NI	25.0
*Aeromonas hydrophila* subsp. *hydrophila* KCTC 2358	NI	NI	NI	NI	NI	NI	NI	NI	NI	NI	NI	NI	NI	≥50.0
*Escherichia coli* KCTC 2441	NI	NI	NI	NI	NI	NI	NI	NI	NI	NI	NI	NI	NI	25.0
*Salmonella typhimurium* KCCM 11862	NI	NI	NI	NI	NI	1000	NI	NI	1000	NI	NI	NI	NI	12.5
Dental plaque bacteria														
*Enterococcus faecalis* KCTC 5289	1000	NI	NI	NI	NI	NI	NI	NI	NI	NI	NI	NI	NI	12.5
*Streptococcus mutans* KCTC 5458	125	NI	NI	NI	NI	NI	NI	NI	NI	NI	NI	NI	NI	0.8
*Streptococcus mutans* KCCM 40105	250	NI	NI	NI	NI	NI	NI	NI	NI	NI	NI	NI	NI	0.8
*Streptococcus oralis* KCCM 41567	500	NI	NI	NI	NI	NI	NI	NI	NI	NI	NI	NI	NI	1.6
*Streptococcus sobrinus* KCTC 5809	250	NI	NI	NI	NI	NI	NI	NI	NI	NI	NI	NI	NI	1.6
*Streptococcus sobrinus* KCCM 11898	250	NI	NI	NI	NI	NI	NI	NI	NI	NI	NI	NI	NI	1.6
*Streptococcus sanguinis* KCTC 5643	500	NI	NI	NI	NI	NI	NI	NI	NI	NI	NI	NI	NI	1.6
Lactic acid bacteria														
*Lactobacillus acidophilus* KCTC 3164	500	NI	NI	NI	NI	NI	NI	NI	1000	NI	NI	NI	NI	3.1
*Streptococcus thermophilus* KCTC 3658	500	NI	NI	NI	NI	NI	NI	NI	NI	NI	NI	NI	NI	1.6

1, *F. vesiculosus* (F85); 2, *F. vesiculosus* (F95); 3, *M. pyrifera* (M85); 4, *U. pinnatifida* (U95); 5, *H. fusiforme*; 6, *K. crassifolia*; 7, *L. japonica*; 8, *S. honeri*; 9, *U. pinnatifida*; 10, *Cap. fulvescens*; 11, *Cod. fragile*; 12, *G. filicina*; 13, γ-carrageenan; and 14, ampicillin. * No inhibition. Data expressed as the mean in duplicate. The samples were tested against the bacterial cell concentration of ca. 5 × 10^5^ CFU mL^−1^.

**Table 3:** Composite monosaccharides in dry fucoidan F85 and F95.

Monosaccharide	F85 (µg mg^−1^) *		(% Ratio)	F95 (µg mg^−1^) *		(% Ratio)
Fucose	471.5	±5.9	(87.4)	521.9	±13.9	(89.7)
Rhamnose	^†^			^†^		
Arabinose	^†^			^†^		
Galactose	33.0	±0.5	(6.1)	29.1	±0.5	(5.0)
Glucose	10.3	±0.6	(1.9)	Nd		
Mannose	5.2	±0.6	(1.0)	11.4	±1.1	(2.0)
Xylose	19.5	±0.5	(3.6)	19.2	±0.3	(3.3)
Fructose	^†^			^†^		
Total	539.5		(100.0)	581.6		(100.0)

* F85, 85% fucoidan from *F. vesiculosus*; F95, 95% fucoidan from *F. vesiculosus*. ^†^ Not detected. Data expressed as the mean in triplicate.

**Table 4:** Change in the weight-average molar masses of fucoidan F85 and F95 before or after heat treatment (121 °C for 15 min).

	Heat Treatment	Retention	Mn *	Mw ^†^	Mp ^‡^	**Mw/Mn ^§^**
		Time (min)	(g mol^−1^)	(g mol^−1^)	(g mol^−1^)	
F85	No	16.40	25,388	74,214	37,800	2.92
	Yes	17.61	7834	13,875	9081	1.77
F95	No	16.19	17,576	62,080	48,763	3.53
	Yes	16.16	38,587	76,113	50,447	1.97

* Number-average molar mass, ^†^ Weight-average molar mass, ^‡^ Peak-frequency molar mass, ^§^ Polydispersity.

## Supplementary Materials

The following are available online at http://www.mdpi.com/1660-3397/16/9/301/s1.
