# Supplementary material for: Antimicrobial and Antibiofilm Activities of Sulfated Polysaccharides from Marine Algae against Dental Plaque Bacteria
Source: Mar Drugs. 2018 Aug 27;16(9):301. doi: 10.3390/md16090301 (PMC6165115; doi:10.3390/md16090301)
Supplement: Supplementary file 1 [file marinedrugs-16-00301-s001.pdf]

Supplementary Information

Article

Antimicrobial and Antibiofilm Activities of Sulfated Polysaccharides from Marine Algae against Dental Plaque Bacteria

Joon-Young Jun [1], Min-Jeong Jung [1], In-Hak Jeong [2], Koji Yamazaki [3], Yuji Kawai [3] and Byoung-Mok Kim [1,*]

Figure S1. Calibration curve and chromatogram of pullulan.

Cirrus GPC Calibration Report

Generated by: GPC
Workbook: C:\Cirrus Workbooks\171106\171106.plw
Method: 171106_POLYSAC

Monday, November 06, 2017 4:22 PM

Calibration: 11/6/2017 4:20:12 PM

Calibration Type: Narrow Standard
Calibration Curve: 12.98 - 0.512x^1

Curve Fit Used: 1

High Limit MW RT: 13.90 mins
High Limit MW: 725144
K: 14.1000
Alpha: 0.7000
FRCF: 1.0000
Residual Sum Of Squares: 0.036603
Coeff. Of Determination: 0.996982
Linear Correlation Coeff.: -0.998490

Low Limit MW RT: 20.82 mins
Low Limit MW: 206
FRM Name:
Flow Marker RT: 0.00 mins
Corrected Sum Of Squares: 12.127510
Standard Y Error Estimate: 0.067641

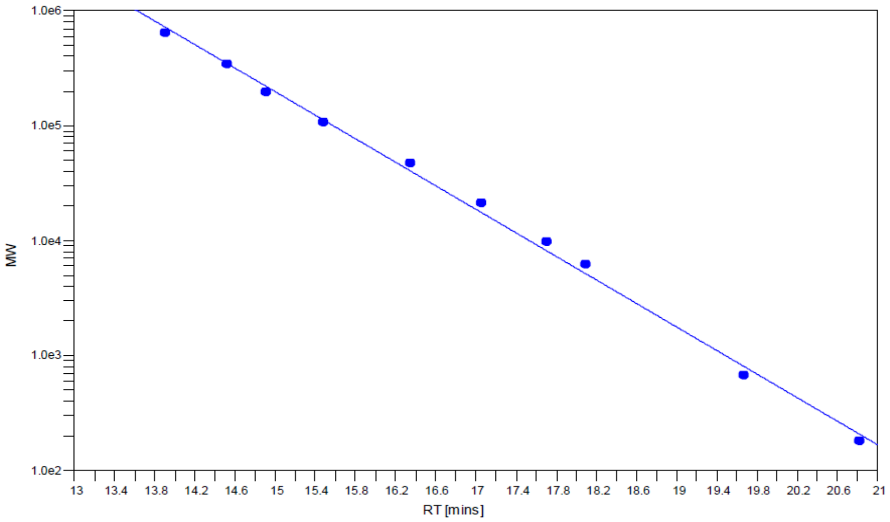

Calibration Points

Pt	Peak Max RT (mins)	MW	Log MW	Point in Use?	Percent Error
1	13.90	642000	5.808	Yes	-12.95
2	14.52	337000	5.528	Yes	-3.69
3	14.90	194000	5.288	Yes	-13.89
4	15.48	107000	5.029	Yes	-4.71
5	16.34	47100	4.673	Yes	14.11
6	17.05	21100	4.324	Yes	16.55
7	17.70	9600	3.982	Yes	14.56
8	18.09	6100	3.785	Yes	14.98
9	19.67	667	2.824	Yes	-20.13
10	20.82	180	2.255	Yes	-14.46

Cirrus GPC Version 3.4.1 Page 1 11/6/2017 4:22 PM

(continued)

Current Chromatogram(s)

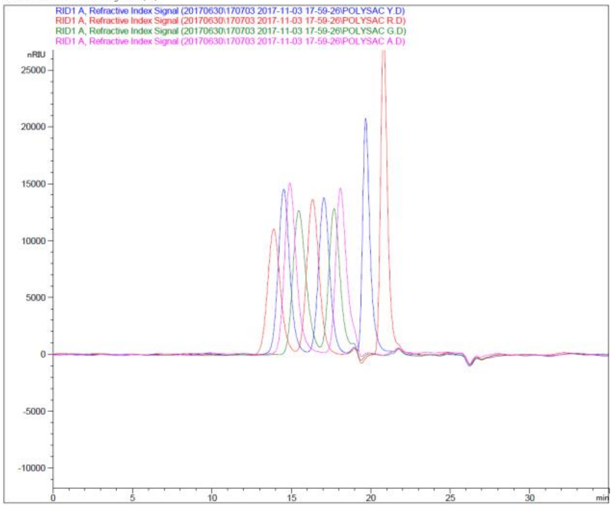

GPC 11/6/2017 3:17:50 PM SYSTEM Page 1 of 1

Figure S2. GPC profile of the fucoidan F85 before heat treatment (121 ºC for 15 min)

Chromatogram report

Sample Name: 85a.d 85A RID1A, Refractive Index Signal
Database name: C:\Cirrus Workbooks\171106\imported-0017.cgm
Method name:171106_POLYSAC
Cahnnel: RI

Data acquisition date and time: 11/8/2017 3:40:03 PM
Calculation date and time:11/6/2017 4:20:12 PM
Acquisition time [min]: 35.006400mins
Calibration type: Molecular Weight

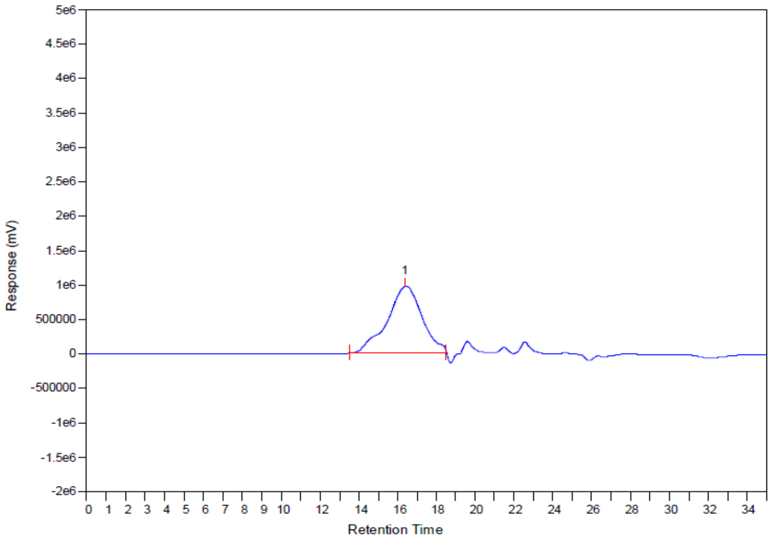

Result of molecular weight calculation (RI)

Peak number:1

Peak start [min]	13.564800	Mn	25388
Peak top [min]	16.401600	Mw	74214
Peak end [min]	18.504000	Mz	204839
		Mz+1	368630
Height [mV]	971074.197018	Mv	62525
		Mp	37800
Area [mV*sec]	122858659.663425	Mw/Mn	2.9232
Area% [%]	100.000000		

(continued)

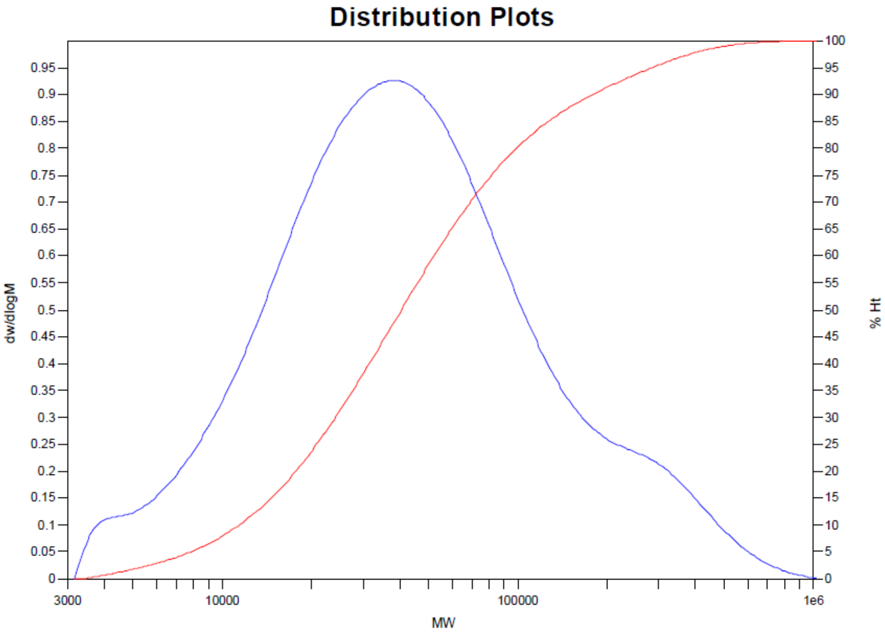
Distribution Plots
dw/dlogM
% Ht
MW
3000
10000
100000
1e6
0
0.05
0.1
0.15
0.2
0.25
0.3
0.35
0.4
0.45
0.5
0.55
0.6
0.65
0.7
0.75
0.8
0.85
0.9
0.95
0
5
10
15
20
25
30
35
40
45
50
55
60
65
70
75
80
85
90
95
100

Figure S3. GPC profile of the fucoidan F85 after heat treatment (121 ºC for 15 min)

Chromatogram report

Sample Name: f85b0.d F85B0 RID1A, Refractive Index Signal

Database name: C:\Cirrus Workbooks\171106\imported-0015.cgm

Method name:171106_POLYSAC

Cahnnel: RI

Data acquisition date and time: 11/8/2017 3:39:20 PM

Calculation date and time:11/6/2017 4:20:12 PM

Acquisition time [min]: 35.006400mins

Calibration type: Molecular Weight

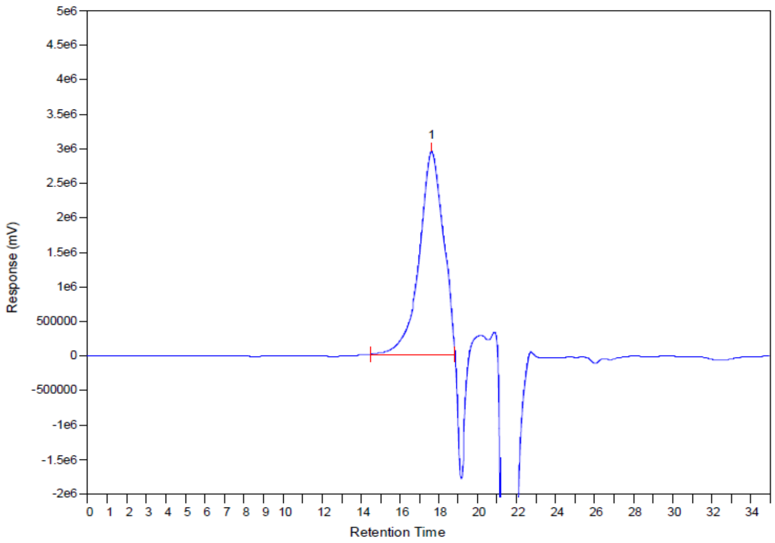

Result of molecular weight calculation (RI)

Peak number:1

Peak start [min]	14.515200	Mn	7834
Peak top [min]	17.611200	Mw	13875
Peak end [min]	18.806400	Mz	36344
		Mz+1	99811
Height [mV]	2941865.282202	Mv	12352
		Mp	9081
Area [mV*sec]	251793209.886428	Mw/Mn	1.7711
Area% [%]	100.000000		

(continued)

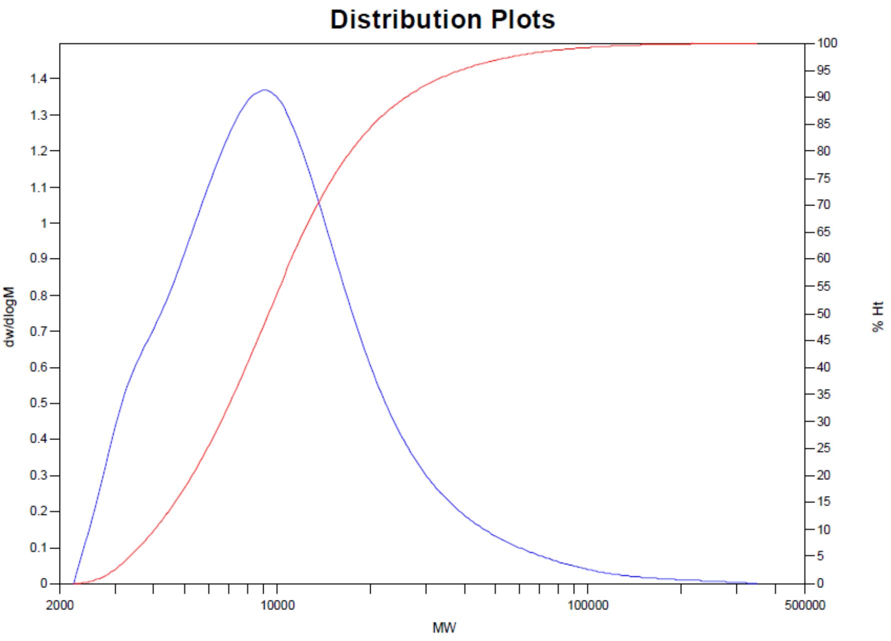

Distribution Plots
dw/dlogM
% Ht
MW
2000
10000
100000
500000

Figure S4. GPC profile of the fucoidan F95 before heat treatment (121 ºC for 15 min)

Chromatogram report

Sample Name: 95a.d 95A RID1A, Refractive Index Signal

Database name: C:\Cirrus Workbooks\171106\imported-0016.cgrm

Method name:171106_POLYSAC

Cahnnel: RI

Data acquisition date and time: 11/8/2017 3:39:58 PM

Calculation date and time:11/6/2017 4:20:12 PM

Acquisition time [min]: 35.006400mins

Calibration type: Molecular Weight

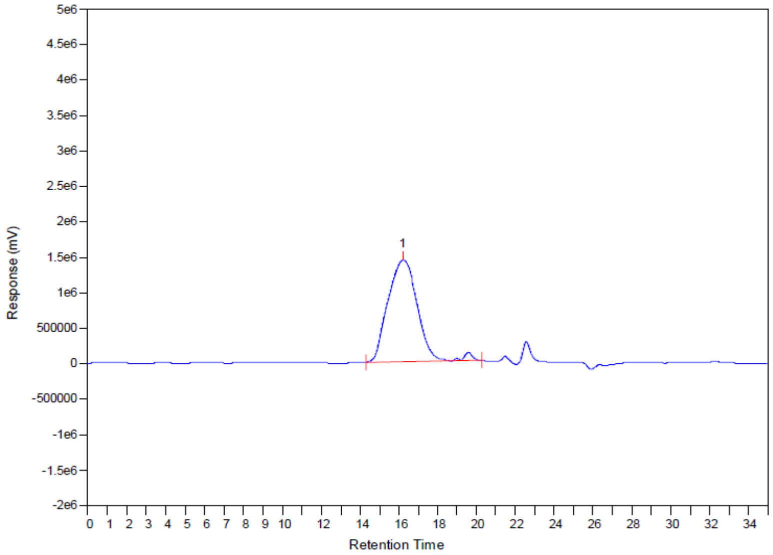

Result of molecular weight calculation (RI)

Peak number:1

Peak start [min]	14.313600	Mn	17576
Peak top [min]	16.185600	Mw	62080
Peak end [min]	20.289600	Mz	102329
		Mz+1	142701
Height [mV]	1439549.390782	Mv	56711
		Mp	48763
Area [mV*sec]	155343404.733925	Mw/Mn	3.5321
Area% [%]	100.000000		

(continued)

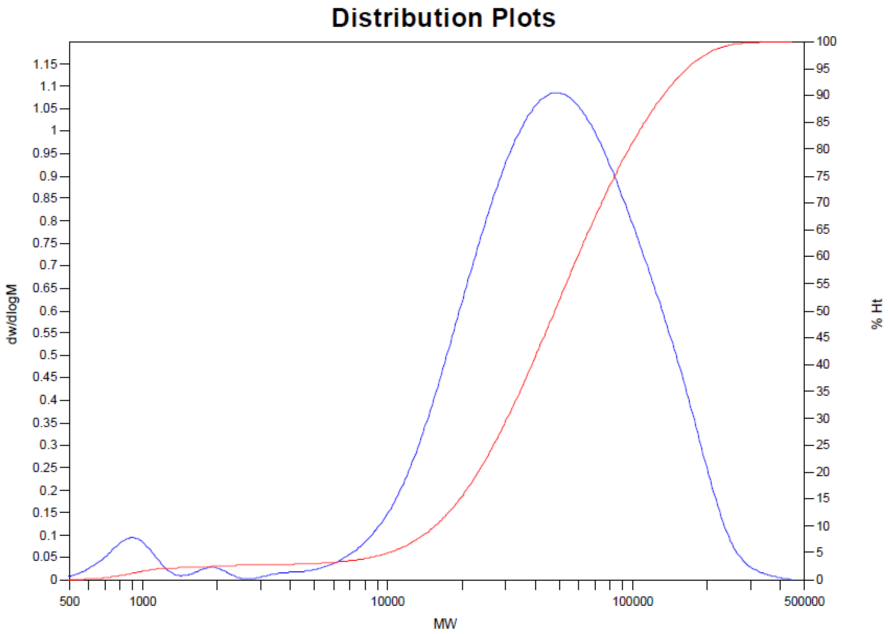
Distribution Plots
dw/dlogM
% Ht
MW
1.15
1.1
1.05
1
0.95
0.9
0.85
0.8
0.75
0.7
0.65
0.6
0.55
0.5
0.45
0.4
0.35
0.3
0.25
0.2
0.15
0.1
0.05
0
100
95
90
85
80
75
70
65
60
55
50
45
40
35
30
25
20
15
10
5
0
500
1000
10000
100000
500000

Figure S5. GPC profile of the fucoidan F95 after heat treatment (121 ºC for 15 min)

Chromatogram report

Sample Name: f95b0.d F95B0 RID1A, Refractive Index Signal
Database name: C:\Cirrus Workbooks\171106\imported-0013.cgrm
Method name:171106_POLYSAC
Cahnnel: RI

Data acquisition date and time: 11/8/2017 3:38:50 PM
Calculation date and time:11/6/2017 4:20:12 PM
Acquisition time [min]: 34.992000mins
Calibration type: Molecular Weight

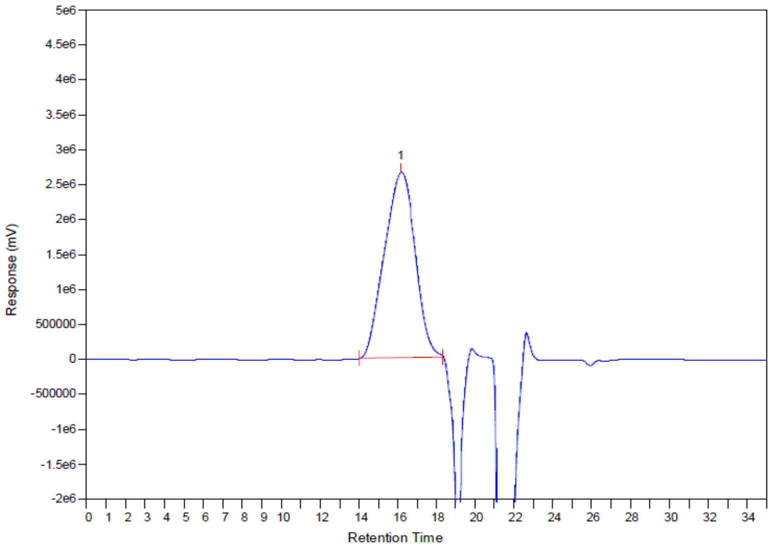

Result of molecular weight calculation (RI)

Peak number:1

Peak start [min]	13.996800	Mn	38587
Peak top [min]	16.156800	Mw	76113
Peak end [min]	18.345600	Mz	136945
		Mz+1	206138
Height [mV]	2657686.912293	Mv	68991
Area [mV*sec]	295778041.155409	Mp	50447
Area% [%]	100.000000	Mw/Mn	1.9725

(continued)

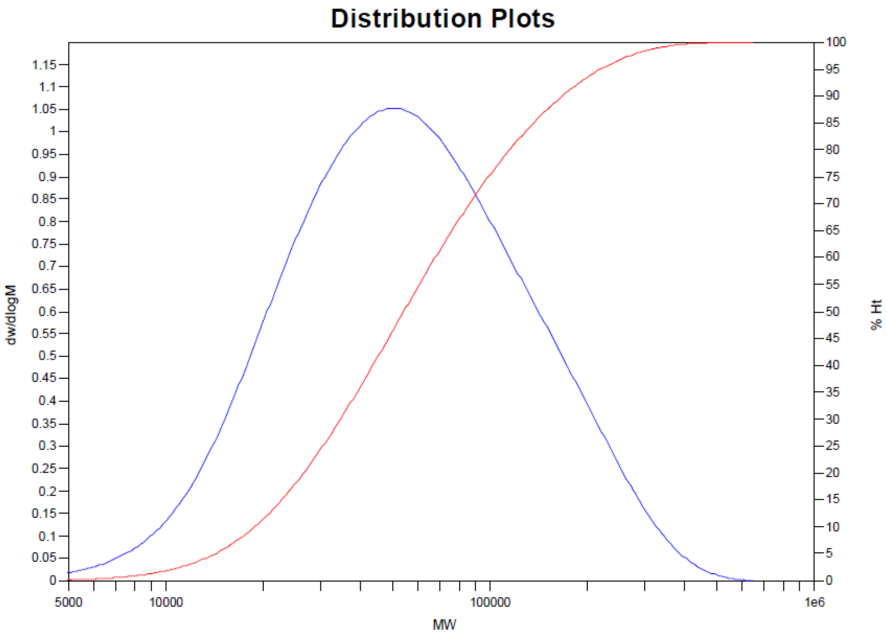

Distribution Plots
dw/dlogM
% Ht
MW
5000
10000
100000
1e6
